# Height Simulation in a Virtual Reality CAVE System: Validity of Fear Responses and Effects of an Immersion Manipulation

**DOI:** 10.3389/fnhum.2018.00372

**Published:** 2018-09-25

**Authors:** Daniel Gromer, Octávia Madeira, Philipp Gast, Markus Nehfischer, Michael Jost, Mathias Müller, Andreas Mühlberger, Paul Pauli

**Affiliations:** ^1^Department of Psychology, Biological Psychology, Clinical Psychology and Psychotherapy, University of Würzburg, Würzburg, Germany; ^2^Department of Psychology, Clinical Psychology and Psychotherapy, University of Regensburg, Regensburg, Germany; ^3^Center of Mental Health, Medical Faculty, University of Würzburg, Würzburg, Germany

**Keywords:** anxiety, fear behavior, virtual reality, presence, immersion, acrophobia

## Abstract

Acrophobia is characterized by intense fear in height situations. Virtual reality (VR) can be used to trigger such phobic fear, and VR exposure therapy (VRET) has proven effective for treatment of phobias, although it remains important to further elucidate factors that modulate and mediate the fear responses triggered in VR. The present study assessed verbal and behavioral fear responses triggered by a height simulation in a 5-sided cave automatic virtual environment (CAVE) with visual and acoustic simulation and further investigated how fear responses are modulated by immersion, i.e., an additional wind simulation, and presence, i.e., the feeling to be present in the VE. Results revealed a high validity for the CAVE and VE in provoking height related self-reported fear and avoidance behavior in accordance with a trait measure of acrophobic fear. Increasing immersion significantly increased fear responses in high height anxious (HHA) participants, but did not affect presence. Nevertheless, presence was found to be an important predictor of fear responses. We conclude that a CAVE system can be used to elicit valid fear responses, which might be further enhanced by immersion manipulations independent from presence. These results may help to improve VRET efficacy and its transfer to real situations.

## Introduction

Exposure therapy is a cognitive behavioral technique for the treatment of anxiety disorders (Abramowitz et al., 2012). Originating from the work of behaviorists who focused on behavioral change (Lang and Lazovik, 1963), exposure therapy has evolved to a multimodal technique, targeting not only behavior but also cognition (e.g., beliefs) and affective states (Schwartz, 1982). Repeated therapeutic exposures to otherwise avoided stimuli (thoughts, objects or situations) allows the patients to experience a decline in anxiety symptoms over time (Foa and Kozak, 1986), offers the possibility to put dysfunctional beliefs to test (Salkovskis et al., 2007), and strengthens self-efficacy in the face of perceived threat (Williams et al., 1985). Similarly, the so-called cue-exposure therapy for substance use disorders and eating disorders, offers these patients the opportunity to experience a decline in craving over time, and strengthens self-efficacy by withstanding drug intake or food bingeing.

Recent research suggests that exposure therapy may be realized in virtual reality (VR), i.e., by exposing patients to computer-generated virtual environments (VEs). This so-called VR Exposure Therapy (VRET; see Bouchard et al., 2012) is convincingly effective for the treatment of phobic disorders (Parsons and Rizzo, 2008; Powers and Emmelkamp, 2008; Opriș et al., 2012; Turner and Casey, 2014; Morina et al., 2015), and first promising results exist for other anxiety disorders as well as substance use disorders and eating disorders (Hone-Blanchet et al., 2014; Freeman et al., 2017; Maples-Keller et al., 2017). For example, VRET for specific phobia involves exposure to VEs which contain the feared stimulus or the feared situation, i.e., spider or height phobics are exposed to virtual spiders or virtual height situations, respectively (see Rothbaum et al., 1995; Shiban et al., 2015). VRET is mostly delivered via a head-mounted displays (HMDs) or a projection-based system which both typically deliver three-dimensional visual and acoustic simulations. Given these first promising results of VRET efficacy, it seems crucial to unravel the underlying mechanisms (Diemer et al., 2016) in order to optimize the treatment and enlarge the field of application.

The efficacy of exposure therapy for anxiety disorders according to the Emotional Processing Theory (Foa and Kozak, 1986) depends on the successful activation of the underlying fear network, as only an activated fear network can be changed with repeated exposures. Successful activation of the fear network by exposure is reflected in initial fear responses, which then habituate within and between exposure sessions. Similarly, the assumption that extinction is the crucial mechanisms of change underlying successful exposure therapy for anxiety disorders (Craske et al., 2012) predicts conditioned fear responses during exposure which decrease with repeated exposure. Based on these theories, any improvement in VRET depends on its ability to elicit initial fear responses in fearful participants.

A psychological construct proposed as an important modulator of the ability of VR to elicit fear is the concept of *presence* (Riva et al., 2015). Presence refers to the sense of “being there” in the VE (Slater et al., 1994; Witmer and Singer, 1998) and can be described by three factors: (a) spatial presence, the strength of the feeling of being inside the VE and interacting directly with it; (b) involvement, the degree how much a person focuses on the VE instead of the real world; and (c) realness, the degree to which experiences within the VE seem consistent with real world experiences (Schubert, 2003, 2009). Although related but theoretically distinct is the concept of *immersion*, a construct describing the objective characteristics of the equipment used to create and display the VR, e.g., number of simulated senses, display size and resolution, framerate and whether stereoscopic presentation is used (Slater et al., 1994; Slater, 1999)^1^. To explicate the distinction between presence and immersion: Presence, on the one hand, describes a feeling that a person in VR can experience (“I feel present in this environment”), thus is a subjective response to a given VE. Immersion, on the other hand, is an objective description of the technological setup used to create and deliver a VE (e.g., using a display with 100° field of view). In general, it is assumed that an increase in immersion realized by technological improvement increases the experienced presence (Cummings and Bailenson, 2015; Diemer et al., 2015), and that an increase in immersion and/or an increase in presence leads to stronger fear responses during VRET and increases VRET efficacy. However, the experimental evidence is ambiguous.

First, there is mixed evidence that an increase in presence enhances VRET efficacy (Wiederhold and Wiederhold, 2000). On the one hand are studies that found no association between presence and treatment outcome. Schuemie et al. (2000) conducted a pilot study on VRET for acrophobia (fear of heights) with six participants and could not find a relationship between presence and therapy outcome. Krijn et al. (2004) compared VRET for acrophobia using either a HMD or a cave automatic virtual environment (CAVE) for stimulus presentation to induce different levels of presence. The CAVE compared to the HMD increased presence, but no effect on treatment outcome was found (Krijn et al., 2004). Price and Anderson (2007) examined effects of presence on treatment outcome of VRET for fear of flying, and also found no significant association. On the other hand, some studies reported that enhanced presence is associated with increased VRET efficacy. Quero et al. (2008) examined patients with different specific phobias, panic disorder with agoraphobia, and patients with eating disorders and revealed a relationship between treatment outcome and subscales of the PRJQ (Presence and Reality Judgement Questionnaire by Baños et al., 2000), specifically the subscales “emotional involvement” and “influence of the quality of software on presence and reality judgment.” Finally, Price et al. (2011) found that the outcome of a VRET for social anxiety was predicted by the “involvement” subscale of the Igroup Presence Questionnaire (IPQ; Schubert, 2003), but not by the other subscales, “spatial presence” and “realism.” Based on these inconsistent findings, we conclude in accordance with Price and Anderson (2007) that presence is a necessary but insufficient requirement for successful VRET.

Second, several but not all studies revealed the assumed positive association between presence and fear responses during VRET (Hodges et al., 1995). For example, Regenbrecht et al. (1998) found a positive correlation between the experienced presence and the fear triggered by virtual height situations. A recent meta-analysis by Ling et al. (2014) incorporating 33 such correlational studies revealed a medium effect size of *r* = 0.28 (95% CI: 0.18–0.38) for the relationship between presence and anxiety during virtual exposures, but also differences between the studied populations: a rather high correlation for specific phobia of the animal subtype and no correlation for social phobia; a higher correlation in clinical anxious compared to non-clinically anxious persons (Ling et al., 2014). However, since such correlational studies do not allow conclusions about causality, additional approaches seem necessary. For example, Bouchard et al. (2008) compared the effects of an anxiety inducing vs. a control VR environment on presence and found increased levels of presence compared to a baseline measure only in the anxiety inducing environment, thus suggesting that anxiety increases presence. In contrast, Peperkorn et al. (2015) studied the temporal dynamics of the presence-anxiety relationship using cross-lagged correlations between multiple VR exposures and concluded that presence predicted fear in the initial exposures while the relationship became bidirectional in later exposures. Therefore, some authors argue that anxiety leads to higher presence (Bouchard et al., 2008), while others assume that higher levels of presence increase the anxiety felt in VEs (Peperkorn et al., 2015), and still others discuss a reciprocal relationship between presence and anxiety (Robillard et al., 2003). In sum, it is still unclear whether presence and anxiety triggered in VEs are causally related and in which direction (see Diemer et al., 2015).

Third, it still remains unclear how immersion modulates presence and/or anxiety. On the one hand, an increase in immersion may increase the experienced presence. Supporting this view, several studies indicate the importance of display and image characteristics (such as field of view, stereoscopy and head tracking; Baños et al., 2004; Krijn et al., 2004; Peperkorn et al., 2015; see Cummings and Bailenson, 2015 for a meta-analysis), as well as haptic cues (e.g., a toy spider) to increase presence (Hoffman et al., 2003; Peperkorn and Mühlberger, 2013). On the other hand, immersion manipulations may increase fear responses triggered by VEs directly (Hoffman et al., 2003; Juan and Pérez, 2009; Peperkorn and Mühlberger, 2013; Peperkorn et al., 2015). For example, in arachnophobia (fear of spiders) treatment, spider models used as haptic cues increased presence and simultaneously increased fear ratings compared to visual-only exposure (Hoffman et al., 2003; Peperkorn and Mühlberger, 2013). Another aspect of immersion which has been manipulated in earlier studies is the use of HMD-based vs. projection-based (e.g., CAVE, Powerwall) VR (Krijn et al., 2004; Juan and Pérez, 2009; Meyerbröker et al., 2011). Both systems have their unique advantages and drawbacks: HMDs offer simpler setup and reduced cost but suffer from screen door effect and cable management, and CAVE systems provide greater field of view and allow the perception of the own body, but at the cost of higher technical effort. We and others conclude that further studies are needed to explore the role of immersion for presence and fear responses and to identify additional presence-increasing methods (Price and Anderson, 2007). Compared to previous VR studies on acrophobia using 4-sided CAVE systems (three walls and floor; Huang et al., 1998; Krijn et al., 2004; Juan and Pérez, 2009; Costa et al., 2014), the present study is the first using a 5-sided CAVE (four walls and floor), which allows participants to turn around and move freely in the given space. Since previous research suggests that allowing movements in the VR height situation compared to standing still increases fear responses for acrophobia (Coelho et al., 2008), we expect a high validity of our VR CAVE system in triggering acrophobic fear. In addition, we realized an immersion manipulation by adding a wind simulation to a virtual height exposure scenario. In acrophobia, no manipulation of immersion by different numbers of applied modalities have been investigated so far, although the situations feared by acrophobics (e.g., a lookout) can contain stimuli of different modalities, e.g., depth perception (visual), wind noise (acoustic) and feeling the wind on the skin (tactile). Thus, the present study is the first to investigate the effects of wind simulation during exposure to a virtual height situation on fear responses and presence.

In sum, this study was designed to examine, first, the validity of the virtual 5-sided CAVE scenario in eliciting fear responses on a verbal and behavioral level. Second, we tested if and how an immersion manipulation (wind simulation) affected experienced presence and fear responses and whether these effects dependent on the participant’s trait acrophobic fear. Finally, we evaluated whether both trait acrophobic fear and presence predict the fear response independently.

## Materials and Methods

### Sample

Volunteers for the study were recruited via public advertisement at the university and a local online platform. The only inclusion criterion was age between 18 and 60 years. The final sample consisted of ninety-nine participants (age: *M* = 22.68, *SD* = 3.84; 65 female participants). Five additional participants were examined but had to be excluded due to technical problems. Based on their Acrophobia Questionnaire (AQ) scores (Cohen, 1977, subscale anxiety, cut-off: 20) participants with a score below/equal to or above 20 were considered low height anxious (LHA; *n* = 44) or high height anxious (HHA; *n* = 55), respectively. Participants received either 6 EUR or course credit as compensation. This study was carried out in accordance with the recommendations of Ethical Guidelines of the German Psychological Society. All subjects gave written informed consent in accordance with the Declaration of Helsinki. The protocol was approved by the Ethics Committee of the Medical Faculty of the University of Würzburg. See Table 1 for group characteristics.

**Table 1:** Questionnaire data.

	LHA		HHA			
	*M*	*SD*	*M*	*SD*	*t*	*p*
AQ Anxiety	10.57	5.80	33.85	12.31	−12.41	<0.001
AQ Avoidance	2.30	2.12	6.22	3.42	−6.99	<0.001
STAI State_t1_	34.71	8.52	39.13	7.37	−2.68	0.009
STAI State_t2_	33.29	7.99	40.72	7.86	−4.53	<0.001
STAI Trait	33.77	7.55	38.69	7.06	−3.30	0.001
SSQ Total	25.93	22.60	44.61	27.06	−3.74	<0.001
IPQ Spatial Presence	4.50	0.66	4.47	0.85	0.22	0.826
IPQ Involvement	3.69	1.32	3.61	1.23	0.32	0.751
IPQ Experienced Realism	3.20	0.98	3.17	1.18	0.14	0.893

*LHA, low height anxious; HHA, high height anxious. AQ, Acrophobia Questionnaire; STAI, State-Trait Anxiety Inventory (*t*_1_ = at the beginning and *t*_2_ = in the end of the experiment); SSQ, Simulator Sickness Questionnaire; IPQ, Igroup Presence Questionnaire*.

### Apparatus

**Figure 1:**
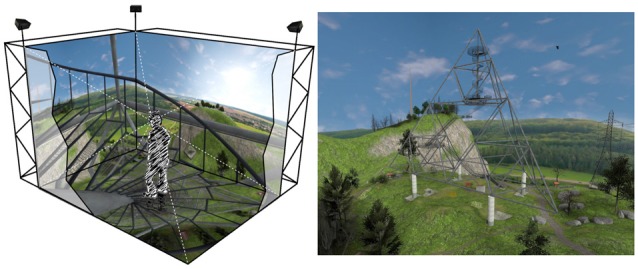
Schematic representation of a participant in the 5-sided Cave Automatic Virtual Environment (CAVE) climbing the stairs of the lookout (left). Screenshot of the VE showing the complete lookout (right). VE developed by VTplus^®^.

The present study was conducted in the 5-sided 3D multisensory PsyCave of the Department of Psychology I of the University of Würzburg, Germany. Rendering of the VE was done with a modification (VrSessionMod 0.5) based on the Source Engine SDK 2007 (Valve, Bellevue, WA, USA) in combination with the CS-Research 5.6 software (VTplus, Würzburg, Germany; see www.cybersession.info for detailed information) for simulation control and data acquisition. The CAVE (see Figure 1; I Space by BARCO, Kuurne, Belgium) has a size of 4 × 3 × 2.95 m. Six projectors (Barco GALAXY NW7) with a resolution of 1920 × 1200 pixels projected stereoscopic images on the four walls and the floor. The resulting resolutions on the different walls were 2016 × 1486 pixels on the front wall, 1627 × 1200 pixels on the door and floor and 1220 × 1200 pixels on the left and right wall. Images were rendered by two computers per projector in order to produce stereoscopic images. To view stereoscopic images, participants had to wear passive interference-filtering glasses (Infitec Premium, Infitec, Ulm, Germany). A 7.1 surround sound system was used for audio presentation. Four fans mounted to the top of the CAVE were used for wind simulation. An active infrared LED system with four cameras (PhaseSpace Impulse, PhaseSpace Inc., San Leandro, CA, USA) was used for movement and orientation tracking. Navigation in the VE was possible by both walking inside the CAVE and using a tracked gamepad (Xbox 360 Wireless Controller, Microsoft, Redmond, WA, USA).

### Virtual Environment

The VE comprised a hilly landscape with a lookout in the center of the scene (Figure 1). The lookout is based on the Tetrahedron in Bottrop (Germany), a steel structure in the form of a tetrahedron^2^ with four platforms at levels of 18 m, 28 m, 35 m and 50 m above the ground. The stairs connecting the platforms are of a see-through metal mesh and the platforms themselves are either of solid steel or the metal mesh.

### Experimental Design and Procedure

The study was based on a 2 × 2 between-subject design with the factors *height anxiety* (low vs. high) and *wind simulation* (fans off vs. on).

Participants gave their informed consent and filled in questionnaires (demographics, AQ and STAI). After being equipped with interference glasses and the gamepad, participants entered the CAVE and completed a training session in a neutral environment to get accustomed to the VR and navigation inside it. Participants were then placed in a mountainous environment with a lookout at the center of the scene (Figure 1) and were asked to walk to the stairs of the lookout to complete several tasks. First, participants climbed the lookout as high as they liked to, indicating with a push on a gamepad button if they did not want to go any higher (behavioral avoidance test, BAT). This was followed by a series of trials where participants were teleported to each level of the lookout and gave their ratings of fear, dangerousness and dizziness (subjective measures of fear). The final task consisted of approaching the railing at the tower’s top-level platform. Again, participants could decide how near they wanted to approach the railing (behavioral measure of fear). After leaving the CAVE, participants filled out another set of questionnaires (STAI State, SSQ and IPQ).

### Measures

#### Questionnaires

AQ (Cohen, 1977). A self-report questionnaire that assesses trait height anxiety on the subscales anxiety and avoidance. The subscale for anxiety comprises of 20 situational items, such as “standing next to an open window on the third floor.” Each item is rated on a seven-point Likert Scale ranging from 0 (“not at all anxious”) to 6 (“extremely anxious”), resulting in a sum score of 0–120. The avoidance subscale consists of the same 20 situational items. Each item is rated on a three-point Likert Scale (“would not avoid doing it,” “would try to avoid doing it” and “would not do it under any circumstances”), resulting in a sum score of 0–40.

*State-Trait Anxiety Inventory* (STAI; Laux et al., 1981). A self-report questionnaire that measures state and trait anxiety. The state anxiety subscale consists of 20 items (e.g., “I am calm”) that are rated on a four-point Likert Scale ranging from “not at all” to “very much so.” Participants are asked to rate the statements according to their present feelings. The trait anxiety subscale also consists of 20 items (e.g., “I am content”) which are rated on a four-point Likert Scale ranging from “almost never” to “almost always.” Participants are asked to rate the statements according to how they feel generally. The range for both scales is from 20–80. The STAI was measured as a control variable.

*Simulator Sickness Scale* (SSQ; Kennedy et al., 1993). A self-report questionnaire that measures simulator sickness, that is symptoms such as nausea, dizziness, headache, or eyestrain, resulting from immersions into VEs. The questionnaire comprises 16 items rated on a four-point Likert Scale ranging from “none” to “severe.” The resulting sum scores are associated to the three factors nausea (e.g., stomach awareness), oculomotor problems (e.g., eyestrain) and disorientation (e.g., vertigo), as well as a total score.

IPQ (Schubert, 2003). A self-report questionnaire that measures the sense of presence in VEs. The questionnaire comprises 14 items rated on a seven-point Likert Scale. The IPQ measures three subscales representing different dimensions of presence. The spatial presence subscale measures a feeling of being inside the VE (e.g., “I felt present in the virtual space”). The involvement subscale consists of items measuring an attentional focus towards the VE (e.g., “I was completely captivated by the virtual world”). The experienced realism subscale measures how real the VE seems to the participant (“How much did your experience in the VE seem consistent with your real world experience?”). One additional item measures a general sense of being in the VE (“In the computer generated world I had a sense of ‘being there‘”). The scores on each subscale have a range of 0–6.

#### Online Ratings

During the experiment, ratings of fear, dangerousness of the situation, and dizziness were assessed by means of Subjective Units of Discomfort Scales (SUDS) ranging from 0 to 100. In addition to the IPQ, spatial presence was assessed with an online rating using the question “To which extent did you feel present in the VE, as if you were really there?” (Bouchard et al., 2005) with a range of 0 to 100.

#### Behavioral Measures

Throughout the experiment, the position of the participants within the VE was tracked continuously. Two measures of behavioral avoidance were derived from the tracking data: how high participants climbed in the first task of the study (BAT) and how close participants approached the railing on the tower’s top-level platform.

### Data Analysis

All statistical analyses were conducted with R 3.2.3 (R Core Team, 2018), and the afex package (Singmann et al., 2018) was used for ANOVA with type 3 sum of squares.

## Results

### Validation of the Virtual Environment

**Figure 2:**
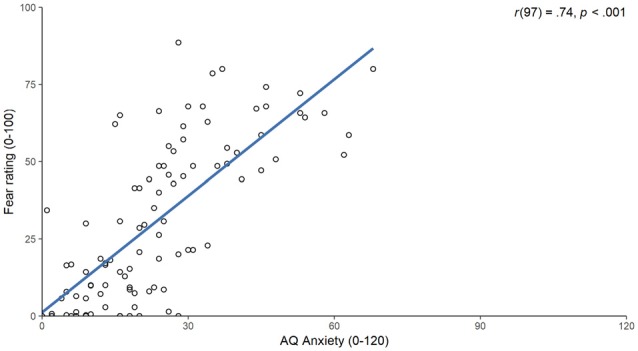
Scatter plot of scores on the Acrophobia Questionnaire (AQ) anxiety subscale (x-axis) and the mean fear ratings (y-axis). The blue line indicates a linear model fitted to the values and the associated correlation is displayed in the top right corner.

To answer the question whether the presented VE was suitable for provoking acrophobia related fear responses, the relationship between a trait measure of acrophobic fear and the fear triggered by the VE was examined. The correlation between the AQ anxiety subscale and the mean fear ratings was *r*_(97)_ = 0.74, *p* < 0.001 (Figure 2). This correlation remained significant after controlling for STAI trait anxiety, as indicated by a partial correlation of *r*_(96)_ = 0.73, *p* < 0.001.

**Figure 3:**
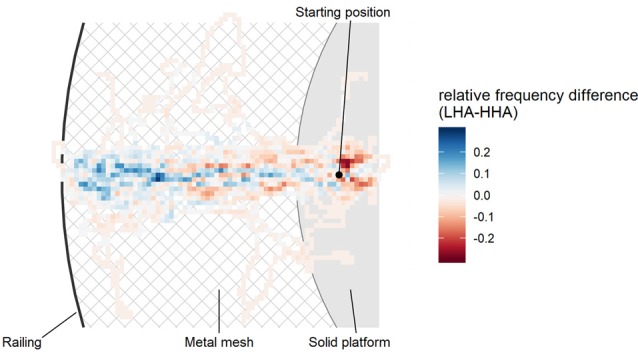
Bird’s eye view on the tower’s top-level platform (50 m). The heatmap shows differences in group movement behavior comparing low height anxious (LHA, blue) and high height anxious (HHA, red) participants. Participants started on a solid platform and had the task to walk over a metal mesh as close to the railing as they wanted. Blue areas indicate that more LHA participants walked there, whereas red areas indicate that more HHA participants were there. The more intense a color is, the greater the relative difference between both groups.

Furthermore, two behavioral avoidance measures, i.e., ability to climb the tower’s top level and walking behavior on the tower’s top level, were evaluated to validate the VE. Since most participants were able to climb to the tower’s top-level platform, we could not conduct a parametric test due to non-normality of the dependent variable. However, a χ^2^-test revealed that significantly more HHA (19 out of 55; 34, 5%) than LHA participants (1 out of 44; 2.3 %) were unable to climb the tower’s top, $\chi_{\left(1\right)}^{2}$ = 13.85, *p* < 0.001. Walking behavior on the tower’s top-level platform was analyzed by comparing the covered distance from the starting position to the railing between groups. The independent samples *t*-test returned that the LHA group (*M* = 2.47, *SD* = 0.53) was able to move significantly closer to the railing than the HHA group (*M* = 2.01, *SD* = 0.81), *t*_(97)_ = 3.22, *p* = 0.002, *d* = 0.65 (see Figure 3).

### Influence of Immersion and Acrophobic Trait Fear on Presence

We tested the effects of immersion by means of wind simulation and acrophobic trait fear on both the online rating of presence and the IPQ scores. For the online rating of presence a two-way ANOVA with group and wind as between factors revealed no main effect of group, *F*_(1,95)_ = 0.46, *p* = 0.500, $\eta_{\text{p}}^{2}$ < 0.01, no main effect of wind, *F*_(1,95)_ = 2.52, *p* = 0.116, $\eta_{\text{p}}^{2}$ = 0.03, and no interaction, *F*_(1,95)_ = 0.90, *p* = 0.345, $\eta_{\text{p}}^{2}$ < 0.01. Descriptively, increased immersion by means of wind simulation somewhat increased the presence ratings in both LHA, *M* = 64.80 (*SD* = 14.82) vs. *M* = 62.32 (*SD* = 18.63), and HHA, *M* = 65.86 (*SD* = 21.37) vs. *M* = 56.00 (*SD* = 18.47); however, effect sizes for this effect were very small ($\eta_{\text{p}}^{2}$ = 0.03). For the IPQ scores, a two-way MANOVA with the four subscales of the IPQ as dependent variables and group and wind as between factors revealed no main effect of group, Wilks’ λ = 0.94, *F*_(4,90)_ = 1.50, *p* = 0.209, no main effect of wind, Wilks’ λ = 0.92, *F*_(4,90)_ = 0.42, *p* = 0.795, and no interaction, Wilks’ λ = 0.98, *F*_(4,90)_ = 2.01, *p* = 0.100.

### Influences of Immersion and Acrophobic Trait Fear on VE Triggered Fear, Dangerousness and Dizziness

**Figure 4:**
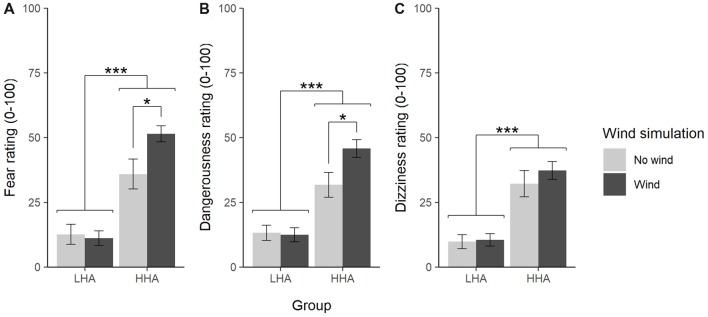
Mean ratings (± standard error) of fear **(A)**, dangerousness **(B)**, and dizziness **(C)** with and without wind simulation. LHA, low height anxious; HHA, high height anxious. *p < 0.05, ***p < 0.001.

The influence of increased immersion by means of wind simulation on mean fear, dangerousness and dizziness ratings was analyzed with three two-way ANOVAs with group and wind as between factors (Figure 4). For the fear rating, the ANOVA revealed a significant main effect of group, *F*_(1,95)_ = 66.06, *p* < 0.001, $\eta_{\text{p}}^{2}$ = 0.41, a marginal significant main effect of wind, *F*_(1,95)_ = 3.20, *p* = 0.077, $\eta_{\text{p}}^{2}$ = 0.03, and a significant interaction, *F*_(1,95)_ = 4.74, *p* = 0.032, $\eta_{\text{p}}^{2}$ = 0.05. Following-up the interaction with *post hoc* comparisons with Tukey’s HSD revealed that increased immersion by wind simulation increased the fear in HHA, *p* = 0.022, but not in LHA, *p* = 0.993 (see Figure 4A). For the dangerousness ratings, there was also a significant main effect of group, *F*_(1,95)_ = 49.41, *p* < 0.001, $\eta_{\text{p}}^{2}$ = 0.34, a marginal significant main effect of wind, *F*_(1,95)_ = 3.21, *p* = 0.076, $\eta_{\text{p}}^{2}$ = 0.03, and a significant interaction, *F*_(1,95)_ = 3.98, *p* = 0.049, $\eta_{\text{p}}^{2}$ = 0.04. Again, Tukey’s HSD revealed a significant difference for wind vs. no wind in HHA, *p* = 0.031, but not in LHA, *p* = 0.999 (see Figure 4B). For the dizziness ratings there was a significant main effect of group, *F*_(1,95)_ = 45.43, *p* < 0.001, $\eta_{\text{p}}^{2}$ = 0.32, but no significant main effect of wind, *F*_(1,95)_ = 0.62, *p* = 0.432, $\eta_{\text{p}}^{2}$ < 0.01, and no significant interaction, *F*_(1,95)_ = 0.36, *p* = 0.551, $\eta_{\text{p}}^{2}$ < 0.01 (see Figure 4C).

### Predicting VE Triggered Fear

The correlation between online presence ratings and mean fear ratings was *r*_(97)_ = 0.31, *p* = 0.002 for the whole sample; within groups, the correlation was significant for the HHA group, *r*_(53)_ = 0.55, *p* < 0.001, but not for the LHA group, *r*_(42)_ = 0.19, *p* = 0.224. A hierarchical regression analysis with fear ratings as dependent variable was conducted to test whether presence explained variance in fear triggered by the VE that was not already explained by the level of trait acrophobia. In the first step, trait acrophobia (as measured by the AQ anxiety subscale) was added to the model. In the second step, presence was added, and then both models were compared. The model with trait acrophobia plus presence explained significantly more variance than the model with trait acrophobia only, *F*_(1,96)_ = 19.99, *p* < 0.001 (see Table 2).

**Table 2:** Results of the hierarchical regression of trait acrophobia and presence on fear ratings triggered by virtual environments (VEs).

	*R*^2^	*AIC*	*B*	*SEB*	*β*	*p*
Step 1	0.54	849.48					<0.001
Intercept			1.16	3.20			0.717
AQ Anxiety			1.26	0.11	0.74		<0.001
Step 2	0.61	832.76					<0.001
Intercept			−22.26	6.00			<0.001
AQ Anxiety			1.24	0.10	0.83		<0.001
Presence			0.38	0.08	0.28		<0.001

*AQ, Acrophobia Questionnaire*.

## Discussion

In this study, participants with different degrees of acrophobic fear (height anxiety) were immersed into a 5-sided CAVE virtual height environment consisting of a 50 m lookout. Immersion was manipulated by adding—besides visual and acoustic simulation—tactile cues (i.e., wind simulation) for half of the participants. While participants were exposed to the VE, we assessed ratings of fear, dangerousness, dizziness and presence, as well as the participants’ avoidance behavior. In addition, we assessed overall experienced presence by means of a questionnaire (IPQ).

### Validity of the CAVE Height Simulation

The assessed verbal *and* behavioral fear responses indicate a high validity for the used VE presented in the CAVE. Compared to LHA participants, the HHA participants experienced more fear, dizziness, and danger in the virtual lookout, and their overall fear level in the VE strongly correlated with a trait measure of acrophobia. Importantly, participants of the HHA group also exhibited enhanced acrophobia-related avoidance behavior, i.e., they more frequently avoided the outlook’s top platform and also remained more distant to the railing of the tower’s top-level platform. These results suggest that the used CAVE system is a valid tool to elicit fear responses related to height. Most previous studies using HMDs reported lower correlations between trait measures of anxiety and VE-elicited fear responses (e.g., Regenbrecht et al., 1998; Mühlberger et al., 2008). In addition, since the CAVE system allows perception of the own body and fairly unrestricted movements this is to our knowledge the first study revealing concordant verbal and behavioral fear responses elicited by a VE which strongly supports the systems validity. Furthermore, the CAVE system seems to be suited to conduct behavioral assessments as a diagnostic instrument adding to self-reported measures (see also Mühlberger et al., 2008).

Since efficacy of VRET likely depends on the adequate activation of the so-called fear network (according to the Emotional Processing Theory, Foa and Kozak, 1986) and/or the presentation of fear-triggering conditioned stimuli (according the inhibitory learning model, Craske et al., 2012) the CAVE system may be an ideal tool for VRET. Indeed, a first study using this CAVE system for VRET for acrophobia revealed promising results (Herrmann et al., 2017).

### Immersion

The observed effect of increased immersion by adding tactile cues (i.e., wind simulation) on reported fear in the VE is in line with previous research using haptic cues (Hoffman et al., 2003; Peperkorn and Mühlberger, 2013). Based on theories on exposure therapy mechanisms of change we suggest that enhancing immersion with tactile cues may help to improve VRET efficacy by providing enhanced emotional responses (Foa and Kozak, 1986) and/or higher dangerousness perception resulting in greater expectancy mismatch if these threat expectancies are violated (Craske et al., 2012). For example, in a fear conditioning study, Brown et al. (2017) showed that greater expectancy mismatch during fear extinction training predicted less fear in an extinction training outcome measure. However, more research on this topic is needed as therapy studies on the relationship between fear activation and therapy outcome are inconclusive (Asnaani et al., 2016).

Interestingly, we did not observe that the immersion manipulation by means of wind simulation had an effect on the experienced presence. This stands in contrast to previous studies reporting an increase in presence by using spider toys as haptic cues in a spider simulation (Hoffman et al., 2003; Peperkorn and Mühlberger, 2013) or adding stereoscopy to a HMD-presented virtual height environment (Mühlberger et al., 2012). One reason might be that our wind simulation was a rather subtle manipulation of immersion. Therefore, a more heavy wind simulation may modulate immersion more explicitly and therefore may increase presence as did noticeable haptic cues, e.g., railings and edges which can be felt with hands or feet, in acrophobia (Schuemie et al., 2000). In any case, the significant effects of our immersion manipulation on elicited fear but not on presence indicate a complex interplay between these variables.

### Presence and Fear

The observed correlation between fear ratings and presence (*r* = 0.31) is in line with previous research (Ling et al., 2014), i.e., increased fear ratings go along with increased presence ratings. Also confirming previous studies (Alsina-Jurnet et al., 2011; Diemer et al., 2016) we observed that this correlation was higher in high anxious compared to low anxious individuals. More importantly, the conducted hierarchical regression models revealed that presence explained variance in fear ratings even when controlling for trait height anxiety. This finding highlights the importance of the concept of presence for fear triggered by VEs (Diemer et al., 2015). However, the current study does not allow conclusions about the causality of the link between presence and fear, and the few previous studies on this research question are inconclusive too (Bouchard et al., 2008; Peperkorn et al., 2015). Both potential causal pathways seem plausible: on the one hand, a higher sense of presence may cause enhanced fear responses because of an increased similarity with real life. On the other hand, experiencing fear in VR might lead to increased presence because the experienced emotional responses increase the experienced realism. Future studies with experimental manipulations are needed to disentangle the relationship between presence and fear. Furthermore, it seems important to elucidate for other disorders whether presence correlates with disorder-relevant responses in VR, e.g., craving responses in exposure to drug cues. First studies on nicotine dependence (Ferrer-García et al., 2010) or eating disorders (Gorini et al., 2010) support this, but research on the causal relationship is lacking.

### Limitations

The present study has some limitations that we have to acknowledge. First, the AQ scores of our sample of HHA participants (*M* = 33.9, *SD* = 12.3) were lower than those of acrophobia samples in previous VRET studies, e.g., *M* = 47.7, *SD* = 9.3 (Coelho et al., 2006), *M* = 59.7, *SD* = 14.1 (Krijn et al., 2004), *M* = 57.1, *SD* = 12.2 (Emmelkamp et al., 2002). However, this is no critical limitation since even stronger effects have to be expected in more anxious samples. Second, we assessed verbal (ratings) and behavioral fear responses to the virtual height situation but not further responses, i.e., physiology (Lang, 1979), cognition (Davis and Ollendick, 2005) and perception (Teachman et al., 2008). Finally, multiple immersion manipulations would have allowed to draw more comprehensive conclusions about the relationship between immersion and fear in VEs.

## Conclusion

The present study demonstrated that a CAVE system is a suitable tool for studying anxiety and fear in ecological valid settings combined with high experimental control. Due to its high validity, this VR setup seems to be an ideal tool to assess different components of the fear response (cognition, physiology, behavior, perception; e.g., Teachman et al., 2008), especially behavioral responses like avoidance and freezing. Results suggest that increase in immersions may enhance VE triggered fear responses, and thus may help to improve VRET. Nevertheless, further studies are needed to disentangle the roles of immersion and presence on both the processes and outcome of VRET.

## Data Availability

The dataset for this study can be found at osf.io/w4e3f.

## Author Contributions

DG, OM, PG, MN, MJ, MM, AM and PP contributed to the study concept and design. Data collection was done by OM, DG and PG. DG performed data analysis and interpretation under the supervision of PP. DG drafted the article and all others provided critical revisions. All authors approved the final version of the article prior to submission.

## Conflict of Interest Statement

PP, AM and MM are shareholders of a commercial company (VTplus GmbH) that develops virtual environment research systems for empirical studies in the field of psychology, psychiatry and psychotherapy. MM is an executive officer of the same company. The remaining authors declare that the research was conducted in the absence of any commercial or financial relationships that could be construed as a potential conflict of interest.

## Footnotes

^1^Witmer and Singer (1998) describe immersion as a psychological state, rather than objective properties of the VR system. According to them, a system is immersive, if it isolates a person from the physical environment. The state of immersion is then defined as “perceiving oneself to be enveloped by, included in, and interacting with an [virtual] environment” (Witmer and Singer, 1998, p. 227). In this article, we will follow Slater’s 1999 definition and use the term immersion for the description of the VR system.

^2^https://en.wikipedia.org/wiki/Tetrahedron_in_Bottrop
